# ‘Gender affirming healthcare’ is not what the family physician needs to know

**DOI:** 10.4102/safp.v67i1.6061

**Published:** 2025-01-13

**Authors:** Janet Giddy, Allan Donkin, Reitze Rodseth

**Affiliations:** 1 First Do No Harm Southern Africa, Cape Town, South Africa; 2 Department of Anesthesiology, School of Clinical Medicine, University of KwaZulu-Natal, Pietermaritzburg, South Africa

In July 2023, the *South African Family Practice* (SAFP) journal published *An introduction to gender affirming healthcare: What the family physician needs to know.*^1^ In this article, Muller et al. promote the Southern African HIV Clinicians Society Gender-Affirming Healthcare Guideline for South Africa (SAHCS GAHG)^2^ which they co-authored. The article recommends that family physicians give cross-sex hormones to ‘Transgender and gender diverse’ (TGD) people and that this should happen at ‘primary care level’.

The terms ‘transgender’ and ‘gender diverse’ are neither in the International Classification of Diseases (ICD-11)-113^3^ nor in the Diagnostic and Statistical Manual of Mental Disorders, Fifth edition, Text Revision (DSM-5-TR).^4^ ICD-11 uses the codes HA60 (Gender incongruence of adolescence or adulthood), HA61 (Gender incongruence of childhood) and HA6Z (Gender incongruence, unspecified). The World Health Organization’s (WHO) rationale (through ICD-11) is that ‘TGD people’ do not have conditions of mental ill-health, and that a mental ill-health classification can cause stigma, an unevidenced claim.^5^ The DSM-5-TR has gender dysphoria as a *diagnosis* using the codes 302.6 (‘Gender Dysphoria in Children’) and 302.85 (‘Gender Dysphoria in Adolescents and Adults’).

Notwithstanding this confusing terminology, there is a global exponential increase in people presenting with gender confusion, gender distress and transgender ideation. The complex aetiology and epidemiology of this phenomenon are poorly understood. It is not explained by the ‘left-handed’ analogy used by Muller et al.

‘Gender affirming healthcare’ (GAHC) has its historical origins in transsexualism and ‘intersex’, and experimentation with body alteration using hormones and surgery. Historical accounts make for disturbing reading.^6^ The lead developer of GAHC is the World Professional Association of Transgender Health (WPATH). The 8th edition of WPATH’s Standards of Care (SOC8)^7^ is their most recent version of their ‘care’ construct. For SOC8, WPATH commissioned a systematic review of cross-sex hormone use. Their own review showed no evidence for efficacy and safety.^8^ Nevertheless, SOC8 goes on to recommend the use of cross-sex hormones.

The World Professional Association of Transgender Health has many affiliates, including the Professional Association of Transgender Health South Africa (PATHSA). The Professional Association of Transgender Health South Africa and Muller et al. promote ‘GAHC’ for ‘TGD people’, who by their own determination, have no pathology. At the ‘TGD’ client’s request, GAHC should be initiated to ‘affirm’ their transgender ideation. Gender affirming healthcare consists of:

- *Social transition* at any age of presentation, including young children.
- *Puberty suppression* in prepubertal children and adolescents.
- *Cross-sex hormones*, including in adolescents and young adults.
- *Body altering surgery*, such as breast and penis amputations, including in adolescents and young adults.

These core interventions, detailed in the SAHCS GAHG and promoted in Muller et al.’s article, create a lifetime dependency on the health system in people with previously healthy bodies.

The SAHCS GAHG draws heavily on WPATH’s SOC7 (SOC8’s predecessor), published in 2012. Neither was compiled using global best practice for guideline development, nor are they based on high-quality evidence. There is much circular referencing. An independent review of the SAHCS GAHG performed by epidemiologists at York University as part of the Cass Review^9^ concluded that the SAHCS GAHG and its recommendations should not be followed.^10^ The Cass Review further concluded that ‘gender affirming’ medical treatments were based on ‘wholly inadequate evidence’ and noticed that young adults between 18 years and 25 years in adult gender clinics are also vulnerable and need safeguarding.^9^

The practice of medicine is a discipline, the cornerstones of which are that for each patient, clinicians need to take a careful history, do a thorough examination, interpret findings into a careful assessment, and then make a considered treatment plan, *guided by good evidence*. This is equally important for people experiencing gender distress. Muller et al. regard this as ‘gatekeeping’.

Thus, GAHC is neither healthcare nor is it a ‘new medical field’ as Muller et al. declare. It is a ‘care’ construct arising out of an ideology that believes that a human can be ‘born in the wrong body’.

The National Department of Health has no policies on providing GAHC at any level. No South African professional medical body no academic institution recognises GAHC as a ‘new field of medicine’. Gender affirming healthcare places children, adolescents and young adults at risk of serious harm. Medical societies, practitioners and scientific journals should foster proper evidence-based clinical practice and health system development. Any training in and practice of GAHC, as formulated and promoted by PATHSA and Muller et al., should *not* happen in mainstream medical care at any level, let alone in the ‘primary health care setting’.

With GAHC not being a ‘new field in medicine’, and not fitting the family medicine paradigm, clinicians should strongly refute the assertion that GAHC is much needed. What is needed, is a careful and evidence-based approach to understanding the complex aetiology, epidemiology, and possible treatment options for people with gender confusion, much of which is still to be established. In the meantime, clinicians need to follow standard and accepted medical and psychological approaches for children, and vulnerable adolescents and adults with gender distress.
