# Corrigendum: ‘Gender-affirming healthcare’ is not what the family physician needs to know

**DOI:** 10.4102/safp.v68i1.6328

**Published:** 2026-03-20

**Authors:** Janet Giddy, Allan Donkin, Reitze Rodseth

**Affiliations:** 1 First Do No Harm Southern Africa, Cape Town, South Africa; 2 Department of Anaesthesiology, School of Clinical Medicine, University of KwaZulu-Natal, Pietermaritzburg, South Africa

In the version of this article initially published, Giddy J, Donkin A, Rodseth R. (2025), ‘*Gender-affirming healthcare*’ *is not what the family physician needs to know* (https://doi.org/10.4102/safp.v67i1.6061)^1^, the disclosure statements required updating. Following post-publication review, the disclosure statements have been amended to clarify the authors’ affiliations and funding.

The amended Competing Interest, Authors’ Contributions, and Funding Information statements appear below.
